# Endoscopic Conservative Treatment of Upper Urinary Tract Urothelial Carcinoma with a Thulium Laser: A Systematic Review

**DOI:** 10.3390/jcm12154907

**Published:** 2023-07-26

**Authors:** Luigi Candela, Eugenio Ventimiglia, Catalina Solano, Marie Chicaud, Stessy Kutchukian, Frederic Panthier, Mariela Corrales, Luca Villa, Alberto Briganti, Francesco Montorsi, Andrea Salonia, Steeve Doizi, Olivier Traxer

**Affiliations:** 1 Division of Experimental Oncology/Unit of Urology, Urological Research Institute, IRCCS Ospedale San Raffaele, 20132 Milan, Italy; 2 Service d’Urologie, Assistance-Publique Hôpitaux de Paris, Hôpital Tenon, Sorbonne Université, GRC n. 20 Lithiase Renale, 75013 Paris, France; 3 Department of Urology, Limoges University Hospital, 2 Avenue M.L. King, 87000 Limoges, France; 4 Department of Urology, Poitiers University Hospital, 2 Rue de la Miletrie, 86000 Poitiers, France

**Keywords:** Thulium, TFL, Thulium:YAG, UTUC, upper tract urothelial carcinoma, conservative treatment

## Abstract

Introduction: Thulium lasers (TLs), namely the Thulium fiber laser (TFL) and the Thulium:YAG (Tm:YAG), are being increasingly adopted for the conservative treatment of upper urinary tract urothelial carcinoma (UTUC). However, to date, the real clinical impact of TLs on UTUC management remains not well-characterized. We performed a review of the literature to summarize the current evidence on TLs for UTUC treatment. Materials and Methods: We performed a systematic review in January 2023 using the Embase and Medline online databases, according to the PRISMA recommendations and using the PICO criteria. Outcomes of interest were: (i) to assess the safety and feasibility of TLs in the treatment of UTUC, and (ii) to evaluate the oncological outcomes in terms of tumor recurrence and conservative treatment failure. Moreover, we described TL characteristics and its interaction with soft tissue. Results: a total of 458 articles were screened, and six full texts including 273 patients were identified. All the included studies were retrospective series. Mean patient age ranged from 66 to 73 years. The indication of a conservative treatment was elective and imperative in 21.7–85% and 15–76% of cases, respectively. Laser power settings varied from 5 to 50 W. No intraoperative complications were reported, and all the procedures were successfully performed. The tumor recurrence rate was 17.7–44%, and the indication to radical nephroureterectomy was 3.7–44% during a follow-up of 6–50 months. Most of the postoperative complications were mild and transient, and ureteral strictures were reported in two studies. Major limitations were the retrospective nature of the studies, the small sample sizes, and the short follow-up. Conclusions: TL is an effective and safe technology for endoscopic UTUC treatment. However, current available literature lacks prospective and multicentric studies with large population sizes and long-term follow-up.

## 1. Introduction

An upper urinary tract urothelial carcinoma (UTUC) is a relatively rare disease with an annual incidence of 1–2 cases per 100,000 inhabitants in Western countries [1]. Until 2013, radical nephroureterectomy has traditionally been the gold standard of care for localized UTUC regardless of tumor characteristics, and endoscopic conservative treatment has been reserved only in cases of imperative indications for kidney-sparing surgery (namely a bilateral UTUC, a solitary kidney, and an impaired renal function) [2]. Based on European Association of Urology (EAU) guidelines, UTUCs are categorized into low-risk and high-risk tumors [3]. Low-risk UTUCs include unifocal disease with size < 2 cm, negative high-grade cytology, low-grade at biopsy, and the absence of an invasive aspect on imaging. Current EAU guidelines strongly recommend managing low-risk UTUCs—and high-risk cases with imperative indications—with kidney-sparing surgery [3].

Endoscopic conservative management of UTUCs is increasingly adopted since improvements in endoscopic equipment and laser technology have allowed successful treatment of low-grade tumors with favorable surgical, oncological, and functional outcomes [4,5]. At present, retrograde intrarenal surgery (RIRS) with a Holmium:YAG laser ablation represents the most widely adopted technique for UTUC kidney-sparing surgeries [6]. Over the last years, the safety, efficacy, and limitations of the Holmium:YAG laser on UTUC management have been widely evaluated [7]. However, despite the proven feasibility and the low rate of complications, RIRS with a Holmium laser is characterized by a non-negligible rate of UTUC recurrence and disease progression in the short and medium term [8]. In this context, improvements in endourologic technologies are needed to further ameliorate endoscopic kidney-sparing surgery outcomes [9].

Recently, the Thulium laser (TL) has promptly spread worldwide due to its advantages compared to the Holmium:YAG laser in urologic endoscopic surgery [10,11,12]. For medical applications, two TL technologies are currently available: the Thulium Fiber Laser (TFL) and the Thulium:YAG laser (Tm:YAG). Data from ex vivo experimental studies showed that TLs may have a lower risk profile and more efficient tissue ablation and coagulation compared to the Holmium:YAG [13,14]. Given this evidence, the TL is increasingly adopted for the conservative treatment of UTUCs with promising results [15]. However, to date, the real clinical impact of TLs on UTUC management remains not well-characterized. Thus, we thought to perform a systematic review (SR) of the literature, with the aim of summarizing the current evidence on TL for UTUC conservative management. Moreover, we aimed to provide an overview of TFL and Tm:YAG operating principles and tissue interaction.

## 2. Materials and Methods 

### 2.1. Study Population and Aims

The population, intervention, comparator, and outcome (PICO) criteria were used to frame the aims of this SR. The population of interest consisted of patients with UTUCs managed conservatively with RIRS; patients with either elective or imperative indications for UTUC endoscopic conservative treatment were considered (P). Thulium laser—either TFL or Tm:YAG laser—represented the evaluated intervention; no dual lasers including TL were considered (I). No other laser or surgical alternative comparators were considered mandatory for the specific purpose of the current review (C). The primary aim was to evaluate the safety and feasibility of TLs in the conservative treatment of UTUCs in terms of surgical complications and complete execution of the surgery. The secondary outcome was to evaluate the oncological outcomes in terms of tumor recurrence and conservative treatment failure (O). Moreover, we decided to describe TL characteristics and its interaction with soft tissue in a narrative modality to summarize the operating principles of this new laser technology and its potential benefits in UTUC treatment.

### 2.2. Literature Search and Data Analysis

**Figure 1:**
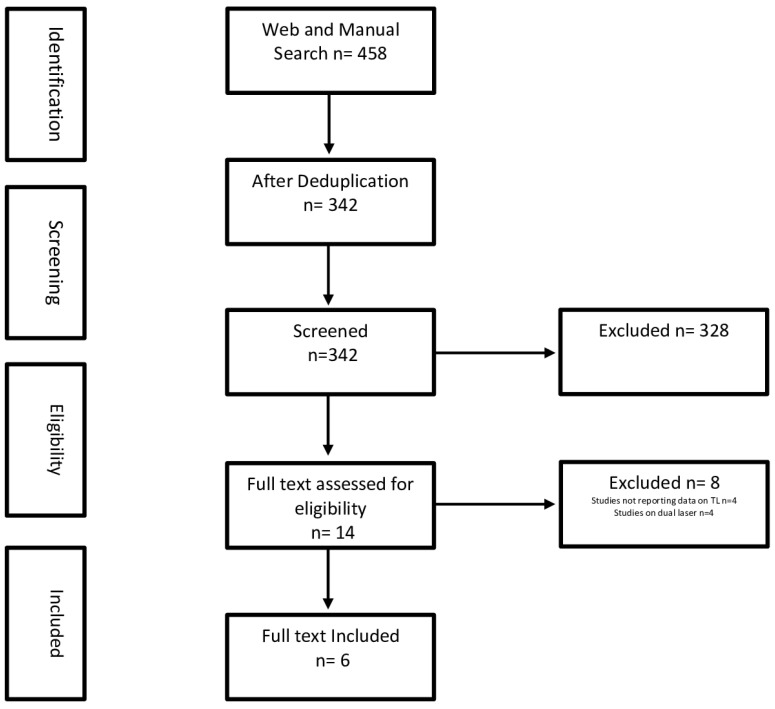
PRISMA (preferred reporting items of systematic review and meta-analyses) flowchart.

A systematic web search was performed on 21 January 2023, according to the preferred reporting items for systematic reviews and meta-analyses (PRISMA) guidelines, with no time restrictions, using the Embase and Medline online databases. The terms “Thulium”, “Thulium:YAG”, and “TFL” were pooled together with the Boolean operator “OR”. The terms “UTUC’’ and “carcinoma” were pooled together with the Boolean operator “OR”. The results were then pooled together with the Boolean operator “AND”. The web Search was implemented by manual search (references of web search included articles). Two authors (L.C. and E.V.) independently screened all items. Disagreements about whether or not to include a study were resolved through consensus or by consultation with a third and senior author (O.T.). Only full-text publications in the English language were considered for the evidence synthesis of the SR. Case reports, editorials, letters, in vitro and experimental animal studies, reviews, and meeting abstracts were excluded from the analysis. Exclusion criteria included studies not reporting data on TLs and studies on dual lasers. Figure 1 shows a flow diagram of the selection process.

A narrative synthesis of included studies was performed. Descriptive statistics were used to summarize the studies’ data and results. Due to the heterogeneity of the study outcomes presentation and the lack of standardization of the follow-up schedule, a meta-analysis of the results was not performed.

## 3. Results

### 3.1. Thulium Laser Characteristics and Soft Tissue Interaction [13,14,16,17,18]

Thulium ion is a rare-earth element with an infrared wavelength emission. For medical laser applications, TL is optimized to emit photons with wavelength of 1940 and 2010 nm for TFL and Tm:YAG, respectively. These wavelengths closely match the infrared absorption peak of water, thus leading to high laser energy absorption of water molecules and hence vapor formation in the target tissue. Cell heating mediated by the laser at different laser settings determines the tissue incision, vaporization, or coagulation through a photothermal effect.

TFL consists of a long and thin coiled silica fiber doped with thulium ions excited by diode lasers during laser activation. The Tm:YAG laser consists of a Yttrium–Aluminum–Garnet (YAG) crystal chemically doped with thulium ions excited via flash lamps in the laser cavity. Therefore, the thulium ions excitation modality for TFL and Tm:YAG is different. On the one hand, TFL is activated by matched diode lasers with minimal heating dissipation; on the other hand, Tm:YAG is activated by pulsed flash lamps that increase temperature during laser activation. Consequently, the latter laser technology needs a water cooling system to avoid overheating, resulting in bigger and heavier laser machines, while TFL systems are air cooled, thus leading to smaller and more practical machines. TL beams can be set with a large spectrum of different energies (0.025–6 J), frequencies (up to 2000 Hz), and pulse shapes, allowing for a huge number of possible of laser settings with a maximum laser power of 60 W. TL beams are transmitted from the laser source to the target tissue through flexible fibers, with small diameters of potentially up to 150 µm, providing endoscopes with maximal deflection and optimal navigation in the urinary tract without affecting laser transmission.

Compared to Holmium:YAG, TL presents a longer pulse length with a lower pick power, resulting in greater cutting precision and hemostatic capacity with a variable tissue carbonization effect, depending on the pulse emission mode. One important advantage of TL in UTUC treatment is its shallow penetration depth in tissue (0.15–0.2 mm), making a precise incision with lower risk of tissue scarring and stricture formation possible.

TFL laser beams are emitted in pulsated emission mode, while Tm:YAG historically operated in a continuous way. Novel pulsated Tm:YAG generators are available, yet without clinical data reported in the literature. This characteristic determines different properties in soft tissue interaction for TFL and Tm:YAG. Particularly, the former causes a smooth incision with excellent coagulation and hemostasis but with more tissue carbonization that may impair tumor visualization during ablation. Conversely, pulsated TFL produces an accurate incision and a fine hemostasis with little carbonization effect.

Given this evidence, TLs showed several advantages compared the Holmium:YAG in terms of soft tissue incision precision and coagulation that allow for excellent cutting and hemostasis during UTUC ablation.

### 3.2. Clinical Data

Titles and abstracts from 458 articles were reviewed, and six full texts were finally selected for this SR (Figure 1). All the included studies were retrospective series (five single centers and one multicenter series), and were published between 2011 and 2022 [19,20,21,22,23,24]. A Thulium:YAG laser and a TFL were used in four [19,21,22,23] and two [20,24] series, respectively. Most of these studies aimed to assess TL safety and effectiveness in conservative UTUC treatment, and reported the short-term complications rate and oncological outcomes. TL was not directly compared with other laser or surgical alternatives in five studies. However, Wen et al. [20] compared oncological outcomes of the 32 patients treated with TL with a control group of 107 patients who underwent radical nephroureterectomy for UTUC in the same center. Moreover, Defidio et al. [19] reported differences between TLs and Holmium:YAG in laser properties through a Likert score for laser performance indicators (e.g., fiber tip stability, fiber tip precision, etc.).

Overall, 273 patients with UTUC were treated conservatively with a TL. Each study presented a relatively small sample size, ranging from 28 to 78 patients (Table 1).

### 3.3. Surgical Technique

Surgical techniques differed slightly among studies. In general, procedures were performed in the lithotomy position under general anesthesia. After urethrocystoscopy and guidewire insertion in the ureteral orifice up to the renal cavities, semi-rigid ureteroscopes were used to manage distal ureteral UTUCs and flexible scopes for proximal tumor treatments. However, Proietti et al. [24] avoided the placement of a guidewire when feasible before the URS (“no touch technique”), and Wen et al. [20] and Bozzini et al. [23] used only flexible scopes for treating UTUCs, regardless the location in the upper urinary tract. Bladder and selective ureteral/renal urine samples were systematically collected for cytological analysis. Ureteral sheath access was often placed in cases of proximal UTUCs. Tumor tissue biopsies for pathological analyses were carried out with stone retrieval baskets or endoscopic forceps. A TL was then used to ablate the residual tumor and for mucosal hemostasis. The diameter of the laser fibers ranged from 200 to 600 μm. Regarding the TL settings, the total power ranged from 5 to 50 W in different studies, with a trend to use less energy and a lower frequency in the ureter compared to the renal cavities to avoid the risk of ureteral damage and potential post-operative stricture formation (Table 1). Most of the surgeons placed a single J or double J ureteral stent after the procedure. Of note, to the best of our knowledge, no series regarding patients treated with a conservative TL for UTUC treatment with a percutaneous surgical approach are available in the literature.

### 3.4. URS II Look and Endoscopic Follow-Up Schedule

EAU guidelines recommend performing a second-look endoscopic procedure within 6–8 weeks after the first URS. Moreover, stringent endoscopic follow-up is mandatory for patients managed conservatively with repeated URS timing, depending on the UTUC risk class [3].

A systematic URS second-look was performed by only Proietti et al. [24], while Defidio et al. [19], Wen et al. [20], and Musi et al. [21] performed early second procedures in cases of non-complete ablation at the first surgery. Nevertheless, in the other series, a second URS was performed at 3 months postoperatively (Table 1).

### 3.5. Patients and UTUC Characteristics

Patients’ mean age ranged from 66 to 73 years old. Patients were predominantly males in all series, expect in the study by Hsieh et al. in which female patients represented 70% of the entire population [22]. Regarding the indication for kidney sparing surgery, conservative treatment was elective in 21.7–85% of cases, while it was imperative in 15–76% of patients. The number of lesions was reported only in three studies; UTUCs were a single lesion in 44–83.8% and multiple lesions in 16.7–56% of patients. Likewise, the tumor location in the upper urinary tract widely varied among studies (Table 2). The mean tumor size was homogeneous among the different series (13–15.3 mm of maximal diameter). At final pathology, most of the UTUCs were low grade, expect in the study by Hsieh et al. in which 74% of tumors were high grade [22] (Table 2).

### 3.6. Surgical and Oncological Outcomes

No intraoperative complications during TL UTUC ablation were reported, and every procedure was completed without technical failures. Only Defidio et al. [19] reported that the median total operative time was 45 (20–90) minutes. In the same study, the authors found that TL was superior to Holmium:YAG in terms of laser fiber tip stability and precision, intraoperative bleeding, mucosal perforation, and operative time for tumors < 1.5 cm [19]. Regretfully, no other data regarding total operative time, laser-on time, and total delivered energy were reported in the other studies.

In the study by Defidio et al. [19], 97% of patients were discharged the day after the procedure. However, Wen et al. [20] reported a postoperative length of hospital stay of 3.6 ± 1.9 days.

One study did not report data on postoperative complications [19]. Three studies reported postoperative complications according to the Clavien–Dindo criteria [21,23,24]. Postoperative complications occurred in 10.5 to 38% of patients. Overall, most of the complications were mild and transient (namely postoperative pain requiring analgesia, hematuria without the need of blood transfusion, urinary tract infection). Regarding major complications, Wen et al. [20] and Hsieh et al. reported four and five [22] developments of ureteral strictures during the follow-up period, Musi et al. [21] performed an hemostatic URS, and Proietti et al. [24] experienced an acute obstructive renal failure that required a double J placement.

Patients’ follow-up ranged from 6 to 50 months among studies. Only Proietti et al. [24] systematically performed a second look procedure within 8 weeks from the first URS, reporting absence of UTUC persistence in 70.4% cases. Overall, UTUCs’ recurrence rate ranged from a minimum of 17.7% of cases with a follow up of 1 year to a maximum of 44% of cases with mean follow-up of 25 months. The longest median follow-up (26.4 months) was reported by Defidio et al. [19], which found a tumor recurrence rate of 37.3%. Conservative endoscopic UTUC treatment failure during the follow-up—namely the indication for a radical nephroureterectomy—was clearly reported in five studies, and it ranged from 3.7 to 39.7% of cases (Table 3). Hsieh et al. [22] reported the development of metastasis in two patients, and cancer-specific death during the follow-up in four (12%) patients. In the study by Wen et al. [20], the authors compared the outcomes of patients treated with TL with a control group of patients treated with nephroureterectomy. The authors found that, despite the lower postoperative creatinine level and the shorter length of hospitalization, patients in the TL group experienced a higher tumor recurrence rate of 21.9% vs. 7.8% in the radical surgery group (*p* < 0.01).

### 3.7. Limitations and Future Directions

This SR has some limitations. First, all the included studies were retrospective series, thus potentially having an impact toward the generalizability of the findings. Second, the sample sizes of the studies were small, and the follow-up times were generally short. Moreover, the follow-up schedule differs among studies. Third, most of the included studies were single-center series and did not use a control group against which to compare TL outcomes. Additionally, the intrinsic differences between TFL and Tm:YAG limit the generalizability of the results. Lastly, the results are presented mainly in a narrative fashion, since a meta-analysis of the data was not performed for the reasons reported in the methods section.

We should highlight that kidney-sparing management itself was identified as a cost-saving alternative to radical nephroureterectomy, with USD 252,272 per patient saved in 5 years [25]. Actually, we did not find studies that analyzed the impact of TL from an economic point of view in the setting of UTUC conservative treatment. Ryan et al. recently reported that TFL has a significantly shorter operative time and decreased cost when compared to the standard Ho:YAG in endoscopic laser lithotripsy [26]. Future studies are needed to investigate the cost effectiveness ratio of TL compared to either to Holmium:YAG or radical nephroureterectomy in patients with UTUCs.

## 4. Conclusions

In the last 10 years, a number of studies on UTUCs managed endoscopically with TLs have been conducted, thus reflecting the growing interest on this laser technology in uro-oncology. Both the TFL and Tm:YAG are safe and feasible laser options to conservatively treat UTUCs. As a whole, TLs showed few complications and an effective tumor ablation rate. Despite these promising results, this laser technology is still characterized by a relatively high short-term rate of UTUC recurrence and conservative treatment failure. The currently available literature lacks prospective and multicentric studies with large population sizes and long-term follow-up. Moreover, there are no studies that directly compare TLs with Holmium:YAG lasers in this setting.

Further multicenter prospective studies with longer follow-ups, larger numbers of patients, and standardized follow-up schedules comparing TLs with other surgical alternatives for endoscopic UTUC management are needed to confirm these findings.

## Figures and Tables

**Table 1:** General features of the included studies.

Authors	Accrual Ys	Country	Aim of the Study	Study Type	Thulium Laser	*n* of Patients	Laser Setting and Fiber	Endoscopic Evaluation after First URS
Defidio et al. [19]	2011	Italy	To evaluate timing and recurrence rates after TL UTUC ablation. To compare TL with Holmium: YAG UTUC ablation	Retrospective, single center	Thulium:YAG	59	10–15 W, 200–365 μm laser fiber	In case of incomplete tumor eradication at first URS; every 3 months during the first year
Wen et al. [20]	2018	China	To assess TFL effectiveness and safety in UTUC treatment	Retrospective, single center	TFL (Quasicontinuous mode)	32	30–50 W, 200–600 μm laser fiber	Every 3 months during the first year and then every 6 months
Musi et al. [21]	2018	Italy	To assess TL effectiveness and safety in UTUC treatment	Retrospective, single center	Thulium:YAG	42	10–20 W, 272–365 μm laser fiber	at 2 months if not radical vaporization; at 3 and 6 months, and then every 6 months if no recurrence
Hsieh et al. [22]	2020	China	to investigate the risk factors of tumor recurrence after TL UTUC ablation	Retrospective, single center	Thulium:YAG	34	5–15 W, 200 μm laser fiber	Every 3 months
Bozzini et al. [23]	2021	Italy	To assess TL effectiveness and safety in UTUC treatment	Retrospective, Multicenter	Thulium:YAG	78	15–30 W, 272 μm laser fiber	Every 3 months for 1 year after first URS
Proietti et al. [24]	2022	Italy	To assess TFL effectiveness and safety in UTUC treatment	Retrospective, single center	TFL (Superpulsed mode)	28	1 J and 10 Hz, short pulse, 200 μm laser fiber	at 2, 6, and 12 months

**Table 2:** Patients and UTUC characteristics.

Authors and Year	N. of Patients	Mean Age (Years)	Elective vs. Imperative Conservative Treatment Indication	Number of Lesions	Tumor Location	Mean Tumor Size (mm)	Tumor Grade
Defidio et al., 2011 [19]	59	66	Elective 85% Imperative 15%	26 (44%) single and 33 (56%) multiple lesions	Renal cavities 50.8% Ureter 22% Multifocal 27.1%	-	-
Wen et al., 2018 [20]	32	69.3	-	-	Renal cavities 12.5% Ureter 87.5%	13	LG 84.4% HG 15.6%
Musi et al., 2018 [21]	42	68	Elective 21.7% Imperative 19% Relative 59.3%	-	Pelvis 31% Proximal ureter 9.5% Middle ureter 12% Distal ureter 35.5% Multifocal 12%	14.3	LG 69.1% HG 9.5% Tis 2.4% Inconclusive 19%
Hsieh et al., 2019 [22]	34	71	Elective 24% Imperative 76%	-	Renal cavities 38% Ureter 62%	-	LG 26% HG 74%
Bozzini et al., 2021 [23]	78	69.2	Elective 76.9% Imperative 23.1%	65 (83.8%) single and 13 (16.7%) multiple lesions	Renal cavities 89.7% Ureter 10.3%	13.5	LG 62.8% HG 37.2%
Proietti et al., 2022 [24]	28	73	Elective 46.4% Imperative 53.6%	16 (57.1%) single and 12 (42.9%) multiple lesions	Pelvis 21.4% Calyces 17.9% Proximal ureter 3.6% Distal ureter 14.2% Multifocal 42.9%	15.3	LG 67.8% HG 28.6% Inconclusive 3.6%

**Table 3:** Postoperative complication and oncological outcomes.

Authors	URS II Look	Overall Complications	Overall Recurrence Rate	Indication to RNU during FU
Defidio et al. [19]	8 (18.6%) patients (performed if UTUC > 1.5 cm)	No intraoperative complications	37.5% of patients (median FU of 26.4 months)	18 (30.5%) patients
Wen et al. [20]	-	4 ureteral strictures	7 (21.9%) patients (FU up to 50 months)	3 (9.3%) patients
Musi et al. [21]	5 (12%) patients (performed if residual disease after first URS)	Clavien–Dindo classification Grade I: 16 (38%) patients Grade II: 15 (35.7%) patients Grade III: 1 (2.4%) patient Grade IV–V: 0	8 (19%) patients (median FU of 26.3 months)	4 (9.5%) patients
Hsieh et al. [22]	-	5 ureteral strictures 4 cancer-specific deaths	44% of patients (mean follow-up of 25 months)	-
Bozzini et al. [23]	-	Clavien–Dindo classification Grade I: 12 (15.3%) patients Grade II: 9 (11.5%) patients Grade III–IV–V: 0	9 (19.2%) patients (mean FU of 11.7 months)	31 (39.7%) patients
Proietti et al. [24]	19 (70.4%) patients: negative 7 (25.9%) patients: positive 1 patient did not undergo a URS II look (performed systematically)	Clavien–Dindo classification Grade I–II: 6 out of 95 procedures Grade III–IV: 1 out of 95 procedures	5 (21.7%) patients (6 months FU) 3 (17.7%) patients (12 months FU)	1 (3.7%) patient

## Data Availability

Please contact the corresponding author.
